# Sigma factor RpoS positively affects the spoilage activity of *Shewanella baltica* and negatively regulates its adhesion effect

**DOI:** 10.3389/fmicb.2022.993237

**Published:** 2022-09-02

**Authors:** Caili Zhang, Jiaqi Chen, Xiaoming Pan, Haimei Liu, Yanlong Liu

**Affiliations:** School of Food Engineering, Ludong University, Yantai, China

**Keywords:** food microbiology, *Shewanella* spp., RpoS, spoilage activity, adhesion, biofilm

## Abstract

*Shewanella baltica* is the dominant bacterium that causes spoilage of seafood. RpoS is an alternative sigma factor regulating stress adaptation in many bacteria. However, the detailed regulatory mechanism of RpoS in *S. baltica* remains unclear. This study aims to investigate the regulatory function of RpoS on spoilage activity and adhesion ability in *S. baltica*. Results revealed that RpoS had no effect on the growth of *S. baltica*, but positively regulated the spoilage potential of *S. baltica* accompanied by a slower decline of total volatile basic nitrogen, lightness, and the sensory score of fish fillets inoculated with *rpoS* mutant. RpoS negatively regulated the adhesion ability, which was manifested in that the bacterial number of *rpoS* mutant adhered to stainless steel coupon was higher than that of the *S. baltica* in the early stage, and the biofilm formed on glass slide by *rpoS* mutant was thicker and tighter compared with *S. baltica*. Transcriptomic analysis showed that a total of 397 differentially expressed genes were regulated by RpoS. These genes were mainly enrichment in flagellar assembly, fatty acid metabolism/degradation, and RNA degradation pathways, which were associated with motility, biofilm formation and cold adaptation. This study demonstrated that RpoS is a primary regulator involved in flagellar assembly mediated biofilm formation and cold adaptation-related spoilage activity of *S. baltica*. Our research will provide significant insights into the control of microbiological spoilage in seafood.

## Introduction

Microbial spoilage of seafood during processing, storage and sales lead to massive economic losses in the aquatic industry (Odeyemi et al., 2020; Zhuang et al., 2021). The psychrotolerant *Shewanella baltica* is a well-known spoilage bacterium frequently detected in chilled fish and seafood (Vogel et al., 2005). Studies indicated that *S. baltica* was the specific spoilage microorganism in raw salmon, chilled shrimp, and large yellow croaker (Macé et al., 2013; Zhu et al., 2015, 2016). *S. baltica* is a gram-negative, motile, rod-shaped bacterium, which is capable of producing H_2_S. *S. baltica* possesses a strong spoilage potential by the formation of spoilage-associated metabolites, such as utilizing trimethylamine-N-oxide as an electronic receptor to produce trimethylamine, dimethylamine and formaldehyde, thus bringing the unpleasant off-flavors (Broekaert et al., 2011; Wright et al., 2019). *S. baltica* decomposed nutrients in fish mainly *via* nitrogen and nucleotide pathways and thereby caused the production of amines, organic acids, etc. (Serio et al., 2014; Lou et al., 2021). Generally, diverse microbiota is discovered on newly caught fish, among which *S. baltica* only accounts for a fraction of the initial microflora, but *S. baltica* dominates and becomes the spoilage microorganism during low-temperature storage (Parlapani et al., 2014). It indicates that *S. baltica* has a robust ability to accommodate rapidly changing conditions. The response of bacteria to different environmental stresses requires complex coordination and overlapping regulatory networks (Zhuang et al., 2021). Adhesion is an important way for bacteria to adapt to the environment, which is a key step in the spoilage process of seafood. *S. baltica* has the potential to attach to seafood and form biofilm on the surface (Zhu et al., 2019). Understanding the environmental adaptation mechanism of *S. baltica* will provide guidelines for the storage of seafood.

Sigma factor is a constituent of bacterial RNA polymerase responsible for recognizing promoters and initiating transcription. Previous studies have shown that the sigma factor regulates a large regulon in Gram-negative bacteria, accounting for about 10% of the genome (Schellhorn, 2014; Wong et al., 2017). RpoS is associated with stress adaptation and response to changeable environments (Dodd and Aldsworth, 2002; Dong and Schellhorn, 2010). The function of RpoS has been widely explored in *Escherichia coli*, *Serratia plymuthica*, and *Vibrio cholerae* (Vidovic et al., 2011; Liu et al., 2016; Wölflingseder et al., 2022), and the results showed that RpoS enhanced their adaptation to various environmental stresses (starvation, acidic pH, oxidative stress, etc.). Recent studies found that RpoS can also regulate virulence factor expression, such as adhesion, biofilm-forming, antibiotic tolerance, etc. Mata et al. (2017) showed that the adherence to epithelial cells of *E. coli* was upregulated by RpoS. RpoS also revealed a positive effect on the expression of T6SS gene and flagellum formation with the increased synthesis of exopolysaccharides in *Y. pseudotuberculosis* (Guan et al., 2015). The role of RpoS played in stress adaptation and toxin factor expression indicated that RpoS might be involved in the environmental adaptation and spoilage ability of *S. baltica*. Feng et al. (2021) demonstrated that sigma factors RpoS/RpoN regulated the stress response and spoilage activity of *S. baltica*. Our previous study indicated that RpoS controlled the stress resistance, quorum sensing and biofilm formation in *S. baltica* (Zhang et al., 2021), however, the detailed genes regulated by RpoS remain unknown.

Recently, many emerging omics methods, such as genomics, metabolomics, and transcriptomics, have been widely applied in the study of food safety with the rapid development of science and technology (Zhao et al., 2020; Li et al., 2021). For instance, the stress responses of *Salmonella enterica* to thermal and essential oils were clarified by metabolomics and transcriptomics (Chen et al., 2022b). Transcriptomics was a feasible technique, which uses the RNA-seq high-throughput sequencing technology to investigate the gene expression level and regulation of food microorganisms at the overall level. Therefore, transcriptomics was applicable to analyze the function of RpoS in *S. baltica.*

For a better understanding of the role of RpoS in *S. baltica*, the difference between *S. baltica* and *rpoS* mutant in spoilage potential and adhesion effect was compared. We further explored the differentially expressed genes (DEGs) in *S. baltica* and *rpoS* mutant using a transcriptome technology. This study aims to clarify the regulatory mechanism of RpoS in *S. baltica*, which will provide new ideas for the control of spoilage microorganisms.

## Materials and methods

### Strains and culture conditions

The *S. baltica* X7 was previously isolated from large yellow croaker. The corresponding *rpoS* mutant was constructed in the previous study (Zhang et al., 2021). Both of these two bacterial species were cultured in LB broth.

### Spoilage activity determination

The spoilage activity of two bacterial strains was evaluated by sterile large yellow croaker fillets. The sterile fish fillets were prepared as described by Macé et al. (2013). The overnight culture of *S. baltica* and *rpoS* mutant were centrifuged and re-suspended in sterile water to OD600 ≈ 0.2. Then 5 ml of the corresponding bacterial suspension was added to 500 ml of sterile water. After that, all the fish fillets were divided into three groups. Two groups were immersed with the bacterial suspension of *S. baltica* and *rpoS* mutant for 10 min, respectively, and the initial bacterial count of fish fillets was approx. 4 log CFU/g. The other group was immersed in sterile water for 10 min as a control (Wang et al., 2017). The fillets were then individually packed in polyvinyl chloride bags and stored at 4 ± 0.5°C. Three fillets from each group were randomly selected for analysis every day. Total viable counts (TVC) were detected as reported by Ozogul et al. (2017). Approximately 25 g of fish fillets were diluted by 225 ml of 0.85% NaCl solution, and the plate count agar was poured onto the dilution and cultured for 48 h at 30°C. The sensory evaluation was conducted based on the studies of Parlapani et al. (2014) and Chen et al. (2022a) with some modifications (Supplementary Table S1). Five trained panelists assessed the fish quality from three aspects: color, texture, and odor. The spoilage level of the fish fillets was scored on a continuous scale from 0 to 10. The total scores of 8–10 points are fresh; 5–7 points are slight spoilage; and 0–4 points are spoilage. The total volatile basic nitrogen (TVB-N) of fish fillets was measured by the Conway and Byrne method using Semi-trace Kevlar Nitrometer (Parlapani et al., 2014). The lightness of fish fillets was determined by a chroma meter (CR-400, Konica Minolta, INC, Japan).

### Bacterial adhesion assays

#### Bacterial adhesion to stainless steel

The adhesion assay of bacterial strains was performed on the stainless steel coupon in LB broth. The 304 stainless steel coupons (10 mm × 10 mm, 1.5 mm thickness) were washed with 75% ethanol and distilled water, and then they were sterilized (Yuan et al., 2018). One milliliter of an overnight culture of *S. baltica* wild-type and *rpoS* mutant was inoculated into a centrifugal tube containing 5 ml LB broth, respectively. Each centrifugal tube contained one stainless steel coupon and was cultured for 24 h at 30°C. The number of colonies attached to stainless steel coupon was determined at 0 and 15 min, 1, 2, 4, 7, 10, and 24 h. The stainless steel coupon was taken out with sterile tweezers and washed with sterile distilled water. The stainless steel coupon was immersed in 5 ml distilled water, handled with ultrasonic for 10 min, and homogenized for 2 min. The determination of the total viable count is the same as described above.

#### Microscopic analysis

For scanning electron microscopy (SEM) observation, the sterilized stainless steel coupons were incubated in LB broth containing *S. baltica* or *rpoS* mutant for 24 h, then the stainless steel coupon was washed with sterile distilled water three times and fixed with 2.5% glutaraldehyde overnight. The coupons were dehydrated by immersing in 30%, 50%, 70%, 80%, and 90% ethanol for 10 min, respectively. Then further dehydration was carried out in 100% ethanol for 15 min twice. Finally, the coupons were dried, gold sputtered and observed using a scanning electron microscope (Hitachi Model SU8010, Japan).

For light microscopic observation, the bacterial cells attached to glass were investigated according to Santhakumari et al. (2018). The sterilized glass slides were plated in LB and cultured for 24 h. Then the glass slides were washed with sterile distilled water and stained with crystal violet for 15 min. The glass slides were examined under a light microscope (Olympus CX22, Tokyo, Japan) after drying.

### Transcriptome samples preparation and analyze

#### RNA extraction and library preparation

Strains of both *S. baltica* and *rpoS* mutant were incubated to OD600 = 1.5, and the bacterial pellet was obtained by centrifugation (4,000 rpm, 15 min). RNA was extracted using a RNeasy Mini Kit (Qiagen, Germany). The rRNA was removed and mRNA was left. The fragmentation was obtained by divalent cations in NEBNext First Strand Synthesis Reaction Buffer. First-strand of cDNA was synthesized with a random hexamer primer. Second strand cDNA synthesis was carried out by DNA Polymerase I and RNase H. The library fragments were purified by an AMPure XP system (Beckman, United States). Afterward, PCR was performed and PCR products were analyzed with the Agilent Bioanalyzer 2100 system. The library preparations were sequenced with an Illumina Novaseq platform.

#### Analysis of RNA-seq

The reads containing adapter, ploy-N (*N* rate > 10%) and low-quality reads were removed and clean reads were collected. The Q20 and Q30 indicated the data were of high quality with values of >98 and 95%, respectively. The clean reads were mapped to *S. baltica* OS678 complete genome in the NCBI database using Bowtie2-2.2.3. Gene expression levels were analyzed by fragments Per Kilobase of transcript sequence per Millions of base pairs sequenced (FPKM; Trapnell et al., 2013). DEGs were analyzed according to the false discovery rate (FDR) values (<0.05), *p* values (<0.05), and the |log2 (fold change)| values (>0.5). The biological functions and metabolic pathways of the DEGs were classified according to the Gene Ontology (GO) and Kyoto Encyclopedia of Genes and Genomes (KEGG) databases. GO terms with adjusted *p* < 0.05 were regarded as significantly enriched by DEGs. The statistical enrichment of DEGs in KEGG pathways was analyzed by KOBAS software.

#### qRT-PCR validation

Bacterial strains were cultured to the end of the exponential phase. RNA was obtained by trizol reagent (Sangon Biotech, China). The EasyScript One-step gDNA Removed and cDNA Synthesis Supermix kit (Transgen, China) was used to compose cDNA. The primers were listed in Supplementary Table S2. TransStart Tip Green qPCR supermix kit (Transgen, China) was used for qPCR amplification. The threshold cycles (CT) were recorded and the relative expression level of genes was obtained by the 2^–△△Ct^ method (Li et al., 2019).

### Data analysis

Each analysis was conducted in triplicate. The results were recorded as the mean ± standard deviation. The significance between samples (*p* < 0.05) was analyzed by SPSS 19.0 software with one-way analysis of variance (ANOVA).

## Results and discussion

### RpoS positively regulated spoilage activity

**Figure 1:**
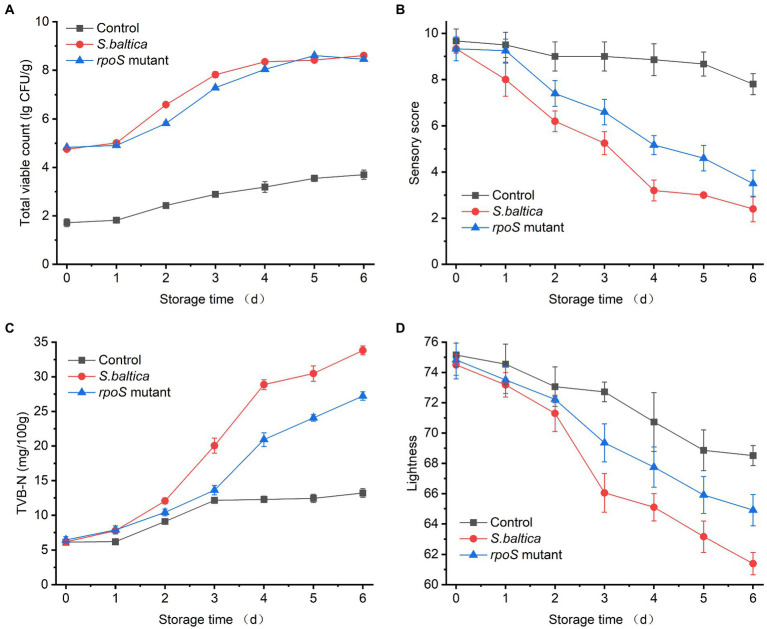
Changes in total viable counts **(A)**, sensory score **(B)**, TVB-N **(C)**, and lightness **(D)** of yellow croaker fillets inoculated with *Shewanella baltica* and *rpoS* mutant during storage at 4°C.

The spoilage activity of bacterial strains was estimated on sterile yellow croaker fillets. The initial viable count for control was below 2.0 log CFU/g. The initial number for fillets that were inoculated with *S. baltica* or *rpoS* mutant was about 4.8 log CFU/g. The number of bacteria increased with storage time, and the growth curves of the two strains were very close, which almost overlapped after 4 days of storage with a viable count of about 8.2 log CFU/g, indicating that RpoS did not affect the growth of *S. baltica* (Figure 1A). Our previous study has shown that the deletion of *rpoS* does not affect the growth of *S. baltica* at neither 30 nor 4°C in LB broth (Zhang et al., 2021). Similarly, the growth of *P. fluorescens* did not change without *rpoS* (Liu et al., 2018). RpoS regulator was first identified as a growth phase-dependent regulator, and it usually plays a role in stress regulation at the stationary phase (Schellhorn, 2014). Therefore, RpoS does not affect the growth of *S. baltica*.

Sensory scores of all samples decreased with the prolonging of storage time because of the production of spoilage metabolite and change in appearance (Figure 1B). TVB-N is one of the typical spoilage metabolites, which is usually used to evaluate the process of fish spoilage. The TVB-N of samples inoculated with *S. baltica* and *rpoS* mutant increased quickly after 3 days of storage, and the group inoculated with *S. baltica* reached a maximum TVB-N value exceeding 30 mg/100 g at day 6 (Figure 1C). The lightness of fish fillets inoculated with *S. baltica* declined quickly compared with that inoculated with *rpoS* mutant (Figure 1D), which was in accordance with the sensory scores. The sensory score of the fish fillets inoculated with *rpoS* mutant was significantly lower than that of *S. baltica* after 3 days of storage (*p* < 0.05), suggesting that RpoS positively regulated the spoilage activity of *S. baltica*. Similar results were also found in *P. fluorescens* and *S. baltica* SB02, the spoilage activities of which decreased when the *rpoS* gene was deleted, which is in agreement with the result in this study. It was inferred that this phenomenon might be attributed to the decrease of extracellular enzyme activity or the down-regulation of quorum sensing in *rpoS* mutant (Liu et al., 2018, 2019; Feng et al., 2021; Hai et al., 2022). The regulatory mechanism of RpoS on bacterial spoilage needs to be thoroughly explored in the future.

### RpoS negatively regulated the adhesion

**Figure 2:**
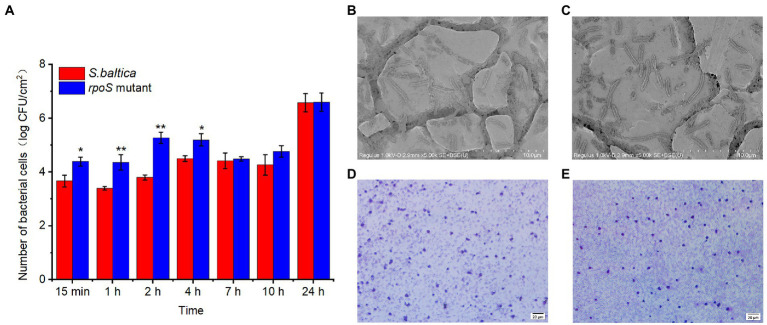
Number of bacterial count on stainless steel coupon, “*” and “**” indicate the significant difference at *p* < 0.05 and *p* < 0.01, respectively **(A)**. Scanning electron microscopy images of biofilm formed on stainless steel coupon by *Shewanella baltica* **(B)**, and *rpoS* mutant **(C)**. Microscopic visualization of biofilm formed on glass slide by *S. baltica* **(D)**, and *rpoS* mutant **(E)**.

Bacteria mediated seafood spoilage is a complex process, involving in adhesion, proliferation, and forming biofilm (Sun et al., 2022). Once bacteria attach to seafood, biofilms will gradually form, thereby causing seafood spoilage (Zhao et al., 2022). The result of bacterial adhesion ability on stainless steel coupon was displayed in Figure 2A. In the early stage of adhesion (0–4 h), *S. baltica* wild type showed a very slow adhesion to the stainless steel, but the number of *rpoS* mutant on stainless steel coupon was higher than that of *S. baltica*. After 2 h of incubation, the number of *rpoS* mutant colonies that adhered to the coupon was nearly 100-fold higher than *S. baltica.* After 24 h, the number of colonies in both strains reached 6.6 CFU/cm^2^, and no significant difference between *S. baltica* and the *rpoS* mutant was observed. It can be observed that the *rpoS* mutant exhibited an earlier and stronger adhesion ability than that of *S. baltica* wild type. For the SEM visualization (Figures 2B,C), similar biofilm morphology of *rpoS* mutant and *S. baltica* on stainless steel coupon was observed, which was in accordance with the attached bacterial count at 24 h. For the glass slides, the biofilm formed by *S. baltica* wild type was relatively loose (Figure 2D), while *rpoS* mutant formed more compact biofilm (Figure 2E). Compared with *S. baltica* wild type, *rpoS* mutant showed earlier adhesion ability on stainless steel coupon and thicker biofilm on the glass slide, indicating that RpoS negatively regulated the adhesion related characteristics of *S. baltica* in a time-dependent and condition-dependent manner.

RpoS usually had a positive response to the changes in the external environment or living state, for example, the motility of *Vibrio cholerae* decreased when the *rpoS* was deleted (Wölflingseder et al., 2022). However, RpoS also manifested some negative regulations. RpoS was found to negatively regulate the swimming motility in *E. coli* and *S. plymuthica* G3 (Ojima et al., 2012; Liu et al., 2016), suggesting that the regulatory mechanism of RpoS may be species-dependent. Consistent with our result, Feng et al. (2021) reported that the deletion of RpoS enhanced the swimming motility in *S. baltica* SB02, and the flagella of the *rpoS* mutant was thicker than that of the wild type observed by transmission electron microscopy. In addition, the biofilm production in *Cronobacter sakazakii* with a defect of *rpoS* only declined in 24 h, and there was no significant difference after 48 h of incubation, revealing that the regulation of RpoS on biofilm formation only showed a delayed effect rather than a complete inhibition (Fernández-Gómez et al., 2020). It revealed that RpoS has a growth-stage dependence on the regulation of biofilm, which provides important insights into the control of *S. baltica* during seafood processing.

### RNA sequencing analysis

In this study, to further investigate the regulatory role of RpoS, a comparative transcriptome analysis of the *rpoS* gene-deficient strain (981 bp segment) and the *S. baltica* wild-type was performed. The total RNA obtained from *S. baltica* and *rpo*S mutant cells were analyzed to identify the DEGs. After filtration, there were on average 7,775,014 and 7,509,599 raw reads for *S. baltica* and *rpoS* mutant, respectively (Supplementary Table S3). A total of 7,638,874 and 7,369,249 high-quality reads from *S. baltica* and *rpoS* mutant, respectively, were mapped on the reference genome of *S. baltica* OS678 (Supplementary Table S3). Approximately 85% of short reads were successfully mapped on the *S. baltica* OS678 genome (Supplementary Table S4).

**Figure 3:**
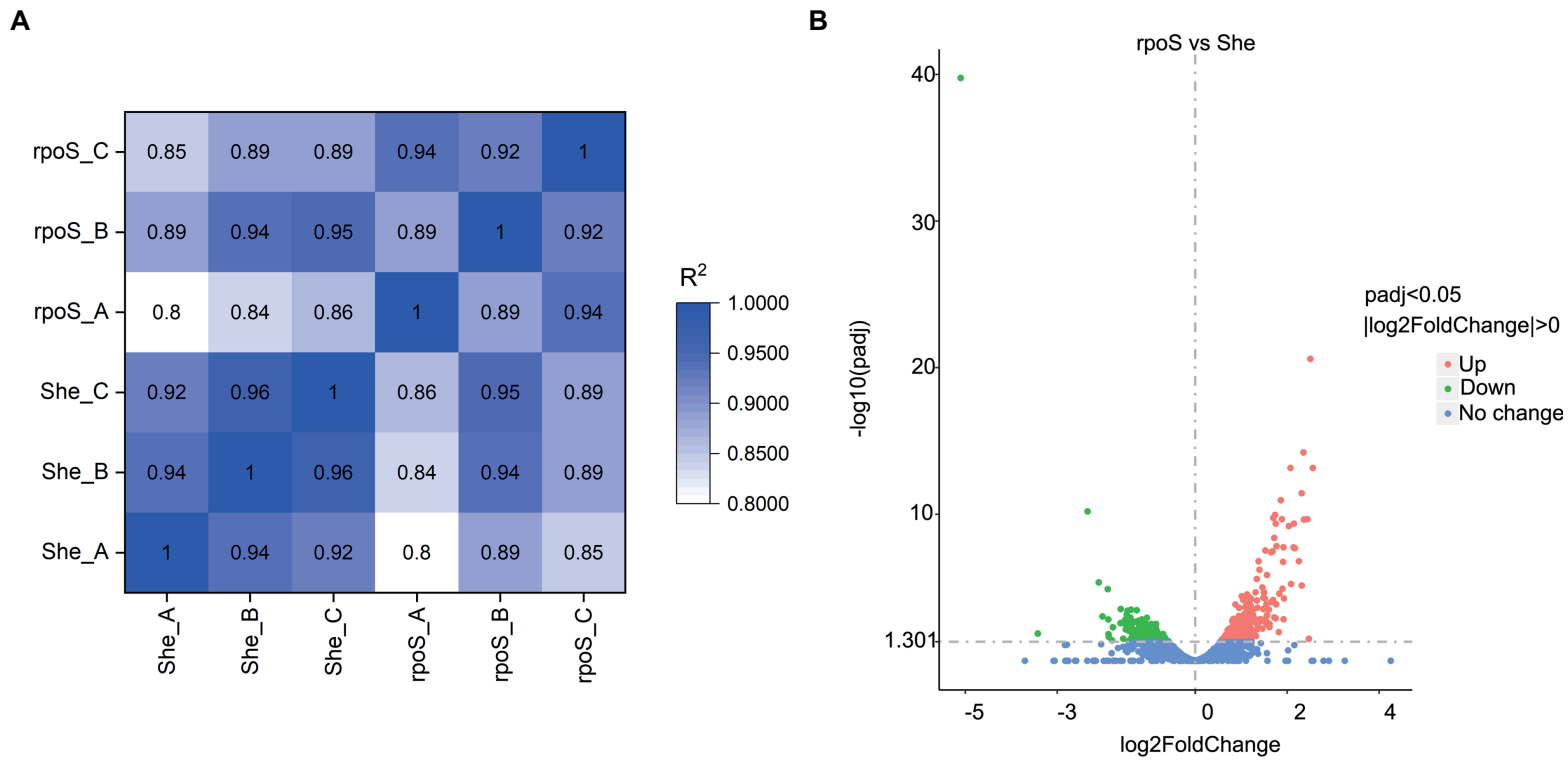
The heat-map of Pearson correlation coefficients based on gene expression levels **(A)**. Volcano plots of different expressed genes **(B)**. “She” indicates *Shewanella baltica* wild type, “*rpoS*” indicates the *rpoS* mutant.

To reduce the impact of sequencing depth and gene length, the corrected FPKM is usually used for describing the gene expression value of RNA-seq. The correlation coefficients indicate the correlations of gene expression between samples. As exhibited in Figure 3A, the squares of the Pearson correlation coefficients (*R*^2^) were all greater than or equal to 0.8, signifying that the expression patterns of genes were very similar between samples (Garber et al., 2011). A total of 397 DEGs was observed, which accounted for almost 10% of the genome (Figure 3B; Supplementary Table S5). The expression of 248 genes (approximately 62%) increased in *rpoS* mutant, while the other 149 genes (approximately 38%) decreased.

**Figure 4:**
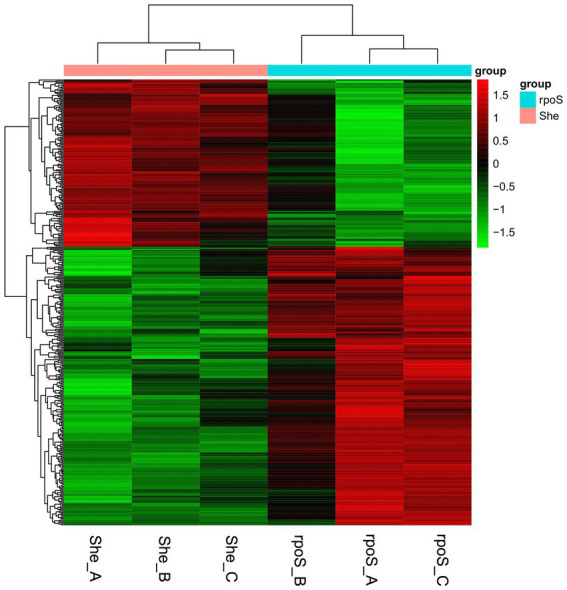
Heatmap of different expressed genes in *Shewanella baltica* and *rpoS* mutant. Each row represents the expression pattern of a single gene and each column corresponds to a single sample. “She” indicates *S. baltica* wild type, “*rpoS*” indicates the *rpoS* mutant.

Hierarchical clustering was performed according to the 397 DEGs, in which the intuitive changes of DEGs between samples were observed (Figure 4). The unsupervised clustering formed two major gene clusters. Cluster 1 (red color) and cluster 2 (green color) indicated the upregulated and downregulated genes, respectively.

### GO analysis

**Figure 5:**
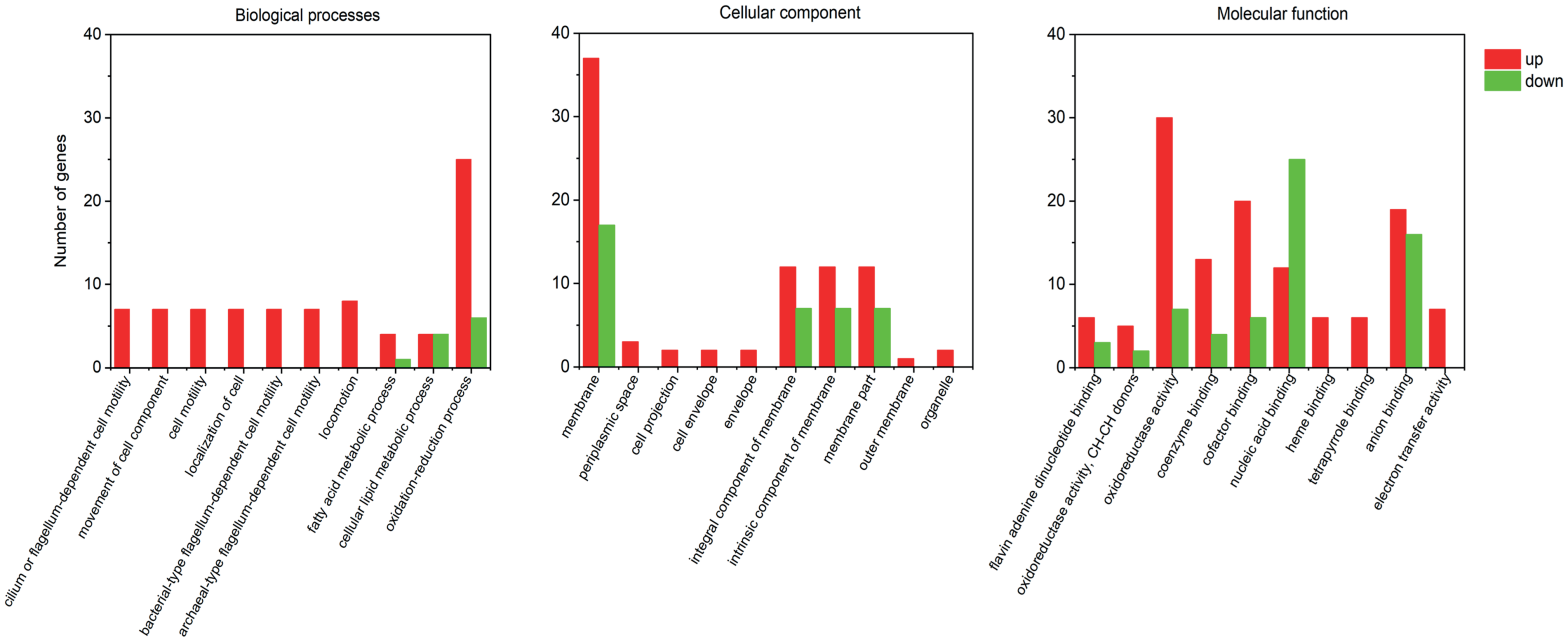
GO enrichment analysis of differently expressed genes.

Gene Ontology enrichment analysis was conducted based on the DEGs. Among the up-regulated DEGs in *rpoS* mutant (Figure 5; Supplementary Table S6), it showed that several categories of biological processes were enriched in the DEGs of *rpoS* mutant, including flagellum-dependent cell motility (GO:0001539, GO:0071973, and GO:0097588), movement of cell component, cell motility (GO:0006928, GO:0048870, and GO:0040011), localization of cell (GO:0051674), and oxidation–reduction process (GO:0055114). As to molecular function, oxidoreductase activity (GO:0016491, GO:0016627), cofactor and coenzyme binding (GO:0048037, GO:0050662), heme binding (GO:0020037), flavin adenine dinucleotide (GO:0050660), tetrapyrrole binding (GO:0046906), and electron transfer activity (GO:0009055) were over-represented. The oxidoreductase activity and cofactor binding were the most represented. The increased motility has been observed in *rpoS* mutant (Zhang et al., 2021), which was mediated by flagellum. The changes in oxidoreductase indicated that RpoS may be involved in the activation of oxidative stress response of *S. baltica*. Therefore, cell motility and oxidoreductase activity were considered to be the main regulation pathways of RpoS.

Among downregulated DEGs in *rpoS* mutant, membrane (GO:0016020) in cellular component, nucleic acid binding (GO:0003676), and RNA binding (GO:0003723) involved in molecular function were representive. In terms of the membrane, the expressions of major facility superfamily (MFS) genes (RS23645, RS43790, and RS29395) were significantly declined in *rpoS* mutant. MFS family transporters are not only related to the absorption of nutrients but also involved in response to external stress. It can be inferred that membrane is one of the main targets of RpoS (Costa et al., 2014).

### KEGG analysis

**Figure 6:**
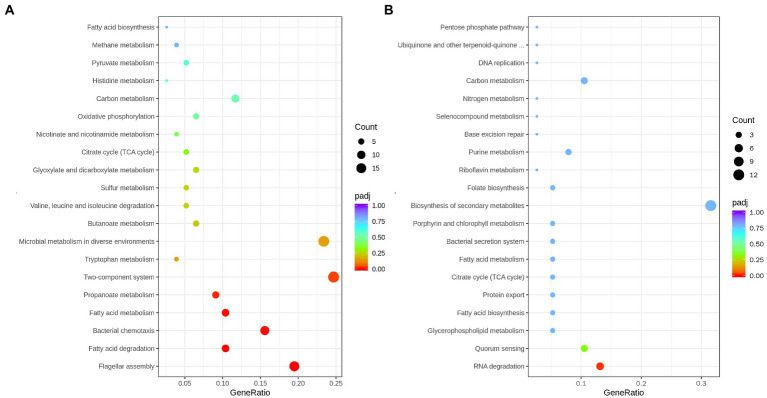
Scatterplot of upregulated genes **(A)**, and down-regulated genes **(B)**, enriched Kyoto Encyclopedia of Genes and Genomes (KEGG) pathway. Enrichment score represents the ratio of the number of upregulated/downregulated genes and the number of all genes in the pathway.

To comprehensively analyze the regulatory function of DEGs, these DEGs were further subjected to KEGG analysis. For the upregulation of DEGs, they were principally focused on flagellar assembly, bacterial chemotaxis, fatty acid metabolism, and two-component system. The downregulation of DEGs were enriched in the RNA degradation and quorum sensing pathways (Figure 6; Supplementary Table S7).

### Flagellar assembly and biofilm formation

**Figure 7:**
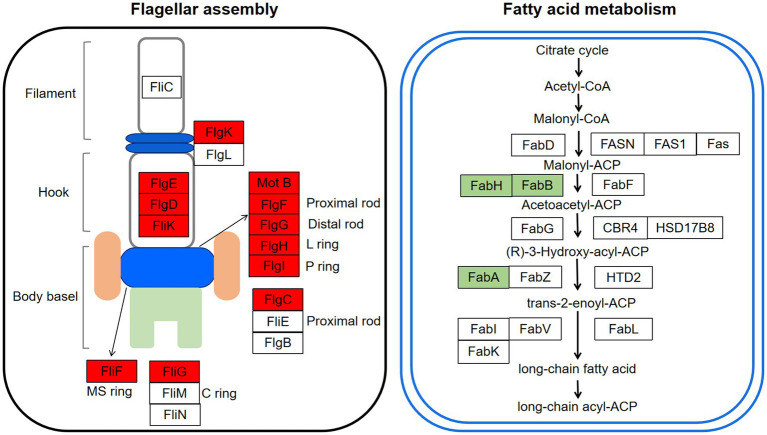
Changes of the expression levels of genes involved in flagellar assembly and fatty acid metabolism of *Shewanella baltica*. The genes marked in red and green represented upregulation and downregulation in *rpoS* mutant.

Flagella is an important motor organ in bacteria. It was reported to have three functional characteristics: motility, chemotaxis, and adhesion, which contribute to responding to the adverse environment quickly and timely for bacteria (Lund et al., 2020). The motility can promote the adhesion of bacteria on host cells, thus realizing infection (Colin et al., 2021). Previous studies indicated that flagella played a crucial role in the adhesion for bacteria, as the *flic* subunit promoted bacteria move to the attachment site in the early stage of adhesion (Sun et al., 2022). It has been observed in our previous study that RpoS negatively regulated the motility of *S. baltica* and the diameter of the *rpoS* mutant increased by 46.6% more than *S. baltica* wild type on the plate (Zhang et al., 2021). The flagella structure consists of three parts: a body basel, hook, and filament (Chilcott and Hughes, 2010). The synthesis and assembly of flagellum are usually involved in more than 50 genes (Chevance and Hughes, 2008). As shown in Table 1, the high expression levels of flagellar body basel-related genes (*flgF, flgG, flgH, flgI, flgC, fliF, fliN*, and *motB*) and flagella hook associated genes (*flgK*, *fliK*, and *flgD*) were found in *rpoS* mutant. The *flgM* and *flgE* showed 1.21 and 0.72 log_2_ (fold change) expression levels, respectively (Figure 7). FlgM protein regulates the transcription level of flagellum-related genes, thus affecting the length and quantity of flagellum. The secretion of FlgM indicates the accomplishment of the Hook-basal body (Wosten et al., 2010). The function of FlgE is to connect the flagellum filament and hook, which changes the angle of flagellum rotation (Shen et al., 2017; Li et al., 2022). Previous studies showed that the enhanced motility of *rpoS* mutant required the RpoN, and about 60% of genes were simultaneously oppositely regulated by RpoS and RpoN (Dong and Schellhorn, 2010). Feng et al. (2021) found that the deficiency of RpoN leads to a decrease in the motility of *S. baltica*, which was owing to the downregulation of flagella assembly-related genes (i.e., *flgF*, *flgC*, etc). The current increased swimming motility of *rpoS* mutant of *S. baltica* might be also affected by the existence of RpoN.

**Table 1:** Representative differently expressed genes in the *Shewanella baltica* wild type and *rpoS* mutant.

Gene ID	Gene	log2 (Fold change)	*p* value	FDR	Gene_description
**Flagellar assembly**					
SBAL678_RS39020	flgM	1.21	3.08 × 10^−6^	1.88 × 10^−4^	Flagellar biosynthesis anti-sigma factor
SBAL678_RS38990	flgD	1.08	9.39 × 10^−5^	2.68 × 10^−3^	Flagellar hook assembly protein
SBAL678_RS38815	fliN	0.95	1.51 × 10^−3^	0.02	Flagellar motor switch protein
SBAL678_RS38975	flgG	0.89	7.80 × 10^−4^	1.32 × 10^−2^	Flagellar basal-body rod protein
SBAL678_RS38970	flgH	0.78	1.43 × 10^−3^	0.02	Flagellar basal body L-ring protein
SBAL678_RS38995	flgC	0.76	9.30 × 10^−4^	1.49 × 10^−2^	Lagellar basal body rod protein
SBAL678_RS38985	flgE	0.72	1.53 × 10^−3^	0.02	Flagellar hook protein
SBAL678_RS38855	fliF	0.72	2.51 × 10^−3^	0.03	Flagellar basal body M-ring protein
SBAL678_RS38980	flgF	0.70	2.25 × 10^−3^	0.03	Flagellar basal-body rod protein
SBAL678_RS38955	flgK	0.65	2.90 × 10^−3^	0.03	Flagellar hook-associated protein
SBAL678_RS46955	flik	1.34	2.11 × 10^−8^	2.62 × 10^−6^	Flagellar hook-length control protein
SBAL678_RS38965	flgI	0.72	2.39 × 10^−3^	0.03	Flagellar basal body P-ring protein
SBAL678_RS30420	pomA	0.81	5.62 × 10^−5^	1.87 × 10^−3^	Flagellar motor protein
SBAL678_RS30425	motB	0.71	2.19 × 10^−3^	0.03	Flagellar motor protein
**Fatty acid metabolism and degradation**					
SBAL678_RS23420	fadB	2.36	8.02 × 10^−13^	2.30 × 10^−10^	Fatty acid oxidation complex subunit
SBAL678_RS23415	fadA	2.15	1.76 × 10^−12^	4.37 × 10^−10^	gene_description
SBAL678_RS37715	fadJ	1.53	1.75 × 10^−10^	2.98 × 10^−8^	Fatty acid oxidation complex subunit
SBAL678_RS37720	fadI	1.20	6.22 × 10^−7^	5.28 × 10^−5^	Acetyl-CoA C-acyltransferase
SBAL678_RS33045	fadD	1.13	9.07 × 10^−6^	4.46 × 10^−4^	Long-chain-fatty-acid--CoA ligase
SBAL678_RS36810	fabA	−0.83	6.25 × 10^−4^	0.01	Bifunctional 3-hydroxydecanoyl-ACP dehydratase/trans-2-decenoyl-ACP isomerase
SBAL678_RS32300	fabB	−1.13	7.00 × 10^−5^	2.21 × 10^−3^	ketoacyl-ACP synthase III
SBAL678_RS32515	acs	1.86	2.08 × 10^−14^	1.11 × 10^−11^	Acetate-CoA ligase
SBAL678_RS43885	acnB	0.88	2.24 × 10^−6^	1.44 × 10^−4^	Bifunctional aconitate hydratase 2/2-methylisocitrate dehydratase
SBAL678_RS44375	prpC	0.71	2.94 × 10^−3^	0.03	2-methylcitrate synthase
SBAL678_RS36490	sucD	−0.70	4.87 × 10^−3^	0.04	Succinate--CoA ligase subunit alpha
**Transport RNA**					
SBAL678_RS36755		−1.90	1.24 × 10^−7^	1.29 × 10^−5^	tRNA-Leu
SBAL678_RS34195		−1.88	1.72 × 10^−3^	0.02	tRNA-Tyr
SBAL678_RS32495		−1.81	3.75 × 10^−3^	0.04	tRNA-Lys
SBAL678_RS38230		−1.62	9.34 × 10^−5^	2.68 × 10^−3^	tRNA-Val
SBAL678_RS36765		−1.62	5.44 × 10^−6^	2.95 × 10^−4^	tRNA-Gly
SBAL678_RS34190		−1.57	2.58 × 10^−3^	0.03	tRNA-Tyr
SBAL678_RS37180		−1.53	5.18 × 10^−5^	1.81 × 10^−3^	tRNA-Ile
SBAL678_RS35065		−1.47	1.83 × 10^−4^	4.55 × 10^−3^	tRNA-Val
SBAL678_RS39045		−1.45	4.48 × 10^−3^	0.04	tRNA-Arg
SBAL678_RS40785		−1.38	1.84 × 10^−4^	4.55 × 10^−3^	tRNA-Leu
SBAL678_RS39860		−1.33	1.20 × 10^−3^	0.02	tRNA-Ser
SBAL678_RS37175		−1.30	1.04 × 10^−3^	0.02	tRNA-Ala
SBAL678_RS31635		−1.29	3.56 × 10^−3^	0.04	tRNA-Pro
SBAL678_RS38195		−1.16	4.71 × 10^−3^	9.06 × 10^−3^	tRNA-Ala
SBAL678_RS40675		−1.16	6.84 × 10^−4^	0.01	tRNA-Met

Biofilm formation is another important flagella-dependent survival model for microorganisms. When the number of bacteria fixed on the surface of host cells increases, the bacteria can secrete extracellular compounds, such as polysaccharides and protein, eventually forming stable biofilms (Voglauer et al., 2022). Zhao et al. (2022) exhibited that acidic electrolysed water disturbed the biofilm formation of *E.coli* by decreasing the nucleotide-related and carbohydrates component. This study revealed that the adhesion and biofilm-forming abilities of *rpoS* mutant were higher than that of *S. baltica* wild type (Figure 2). RNA degradation declined in *rpoS* mutant, while protein and carbohydrate metabolism had no significant change (Figure 6). We inferred that flagella assembly and RNA degradation might play a more important role in biofilm formation than nutrient metabolism The present study indicated that RpoS negatively regulated the flagellar-associated gene expression and biofilm formation. Therefore, RpoS-mediated flagellar assembly is one of the most crucial features of *S. baltica* in environmental adaptability.

### Fatty acid metabolism

Fatty acid metabolism and fatty acid degradation are also important pathways regulated by RpoS (Figure 6). Fatty acids are the main components of the bacterial cell membrane. Fatty acid metabolism has been identified to be closely related to bacterial adaptation to the cold environment because the low temperature can reduce bacterial membrane fluidity and growth rate by adjusting the composition of fatty acids, especially the unsaturated fatty acids (UFAs) (Wang et al., 2020). In the fatty acid metabolism pathway, FabA plays a key role in the synthesis of UFAs. FabA is a bifunctional enzyme that can dehydrate β-hydroxy-decanoyl-ACP and isomerize trans-2-decenoyl-ACP in order to stimulate UFAs synthesis. While *fadA, fadB*, *fadD*, *fadE*, *fadF*, *fadI*, and *fadJ* are the essential genes in the fatty acid degradation pathway (Feng and Cronan, 2011). This study indicated that *fabA* was down-regulated by RpoS, while the expression of *fadA, fadB*, *fadD*, *fadI*, and *fadJ* were upregulated (Table 1), indicating that the deficiency of *rpoS* decreased the synthesis of UFAs in *S. baltica*. FabH is β-Ketoacyl-ACP synthase, which is the initiation factor of the carbon chain extension cycle, and *fabH* was down-regulated in *S. baltica* (Figure 7). Phosphatidylglycerol phosphate synthase (PgsA) catalyzes the primary reaction of phosphatidylglycerol biosynthesis, which was down-regulated in the *rpoS* mutant. *Shewanella baltica* is one of the most common specific spoilage bacteria in refrigerated fish and seafood. The strong adaptability to low temperature contributes *Shewanella* to maintaining a great growth state under refrigerated conditions. Initiation factor 1 (IF1) is encoded by *infA*, which is associated with the initiation step of protein synthsis in bacteria. The *infA* gene was positive-regulated by RpoS in *S. baltica* strain (Supplementary Table S5). Ko et al. (2006) reported that the expression of IF1 increased when *E. coli* cells were treated with cold shock, which is in accordance with our result. The deficiency of RpoS decreased the spoilage ability in *S. baltica* (Figure 1), which may be related to the decline of cold adaptation. Wang et al. (2020) reported that cell membrane UFAs-mediated cold adaptation was closely related to the spoilage potential. However, there are few reports on the relationship between RpoS and cold adaptation. Therefore, the cold adaptation regulatory mechanism regulated by RpoS needs to be further studied, which will contribute to clarifying the spoilage characteristics of *Shewanella* spp.

### Two-component regulatory systems

Microorganism can assemble some necessary genetic circuits to sense external stimuli and respond to environmental stress. Two-component regulatory system (TCRS) is one of the most important signal transduction systems that allow bacteria to respond to stress conditions and improve their survival (Nguyen and Hong, 2008). The present study showed that TCRS was upregulated in the *rpoS* mutant of *S. baltica*. Bacterial TCRS is involved in the regulation of bacterial chemotaxis, morphological differentiation, nutrient metabolism, drug resistance, and many other physiological processes. Li et al. (2022) showed that the expression of *rpoS* can be activated by the two-component system of TorR using transcriptomics, while the effect of RpoS on TCRS was rarely studied. The up-regulated TCRS in the *rpoS* mutant indicated that TCRS is involved in the stress regulation of RpoS to *S. baltica*, but the detailed mechanism needs to be further explored.

## Transport RNA

The tRNA mainly participates in the process of protein translation. Table 1 showed that 15 kinds of the transfer RNA (tRNA) were downregulated, indicating that RpoS contributes to the tRNA-mediated protein synthesis. Studies revealed that translation regulation is an important mechanism for bacteria to respond to stress quickly, and the dynamic adjustment of the total amount of tRNA is a critical regulation means for bacteria to deal with the harsh environment. Zhong et al. (2015) reported that *E. coli* slowed down the rate of translation extension by downregulating the expression of tRNA, which makes cells promptly adapt to oxidative stress.

### Quorum sensing

Quorum sensing (QS) is a mechanism that microorganisms communicate to sense chemical signaling change, thus mediating the toxin factors expression, such as biofilm formation and extracellular enzyme production (Mukherjee and Bassler, 2019). Figure 6 showed that the expression of QS pathway decreased in the *rpoS* mutant. Consistent with this study, our previous study demonstrated that QS signals cyclo-(L-Pro-L-Leu) and cyclo-(L-Pro-L-Phe) declined in *rpoS* mutant, and the expression levels of QS sensing genes *luxR* were also declined in *rpoS* mutant (Zhang et al., 2021). Similar phenomena were also found in *P. fluorescens* and *S. baltica* SB02, in which the acyl homoserine lactone and diketopiperazines QS signaling decreased in the deletion of *rpoS* mutant (Liu et al., 2018; Zhang et al., 2021), indicating that RpoS usually positively regulated the QS. Therefore, QS is expected to become a new target to control the spoilage caused by *S. baltica*.

### Others

Cold shock protein is an important protein that affects the ability of bacteria to adapt to low temperature. Cold shock DNA-binding domains (RS31005) were downregulated in the *rpoS* mutant (Supplementary Table S5). Cold shock protein can be used as molecular chaperones to bind with single-stranded DNA or single-stranded RNA, which prevents DNA or RNA from forming secondary structures at low temperature, thereby promoting transcription and translation (Mihailovich et al., 2010). DNA gyrase B and *parE* (RS27555), which were the targets of antibiotics in Gram-negative bacteria, were downregulated in *rpoS* mutant. Bacterial DNA gyrase is the basic enzyme involved in the formation of DNA superhelix, and the decrease of gyrase related genes usually causes antibiotic resistance (Jogula et al., 2020). It suggests that RpoS take part in the regulation of antibiotic resistance of *S. baltica*. Previous studies indicated that the *rpoS* gene was involved in tolerance to antibiotics in *Pseudomonas aeruginosa* during the stationary phase (Keiji et al., 2010), and the deficiency of *rpoS* in *Salmonella Enteritidis* decreased the tolerance to essential oils (Cariri et al., 2019), which supports our conclusion.

Danhorn and Fuqua (2007) described that flagellum generally guides bacteria colonized on food-contact surfaces in the initial adhesive stage, then the flagella declined with the bacterial proliferation. Food gradually spoiled with the bacteria adapting to the environment and secretion of extracellular enzymes (Hai et al., 2022). Previous studies have confirmed that fatty acid synthesis and QS can enhance the growth and spoilage ability of *S. baltica* (Zhu et al., 2015; Wang et al., 2020). In this study, the fatty acid synthesis and QS pathways were downregulated in *rpoS* mutant, and its spoilage ability also decreased, but the gene expression related to flagellum assembly increased. Therefore, we speculated that RpoS might improve the survival strategy and spoilage activity through early adhesion mediated by flagella assembly pathway as a crucial target. Overall, RpoS is an important regulator involved in the environmental adaptation and spoilage potential of *S. baltica*.

### qPCR validation

**Figure 8:**
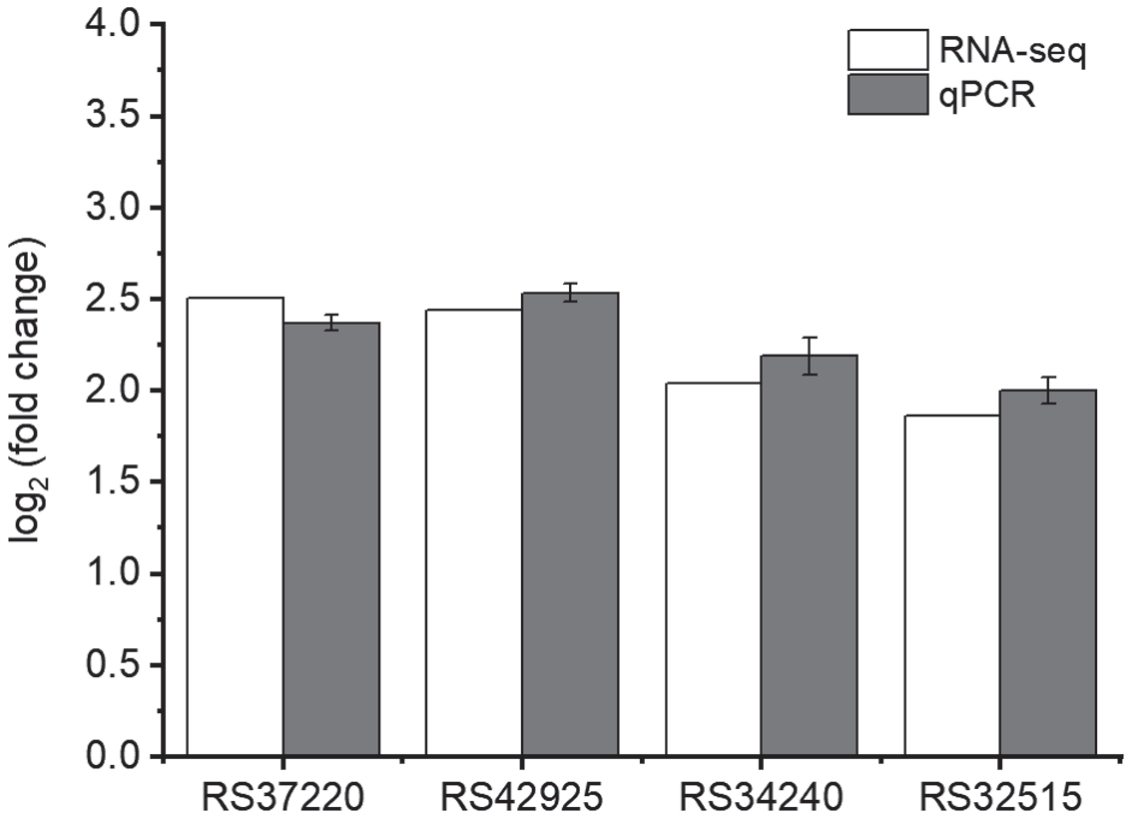
Validation of RNA-seq data of selected genes by qPCR.

To validate the RNA-seq data, qPCR was conducted on four genes (RS37220, RS42925, RS34240, and RS32515) regulated by RpoS. These selected genes showed up-regulation in *rpoS* mutant compared with *S. baltica*. The expression levels were very close in RNA-seq and qPCR results (Figure 8), which indicated that the transcriptome results were reliable.

## Conclusion

This study revealed that RpoS positively regulated the spoilage activities of *S. baltica* while negatively regulated the adhesion and biofilm formation. Transcriptome analysis indicated that large numbers of genes were regulated by RpoS, mainly including flagellar assembly, fatty acid metabolism, two-component regulatory systems, and RNA degradation. These biological pathways might be involved in colonization, stress response, cold adaptation, and spoilage potential of *S. baltica*. Meanwhile, some other pathways such as quorum sensing were also mediated by RpoS, indicating that RpoS has a wide range of regulatory functions in cell processes. The transcriptome results are reliable through qPCR validation. The present results deepened our understanding of the bacterial spoilage mechanisms. RpoS might be used as a new target for food preservation in the future.

## Data availability statement

The datasets presented in this study can be found in online repositories. The names of the repository/repositories and accession number(s) can be found at: NCBI BioProject—PRJNA858939.

## Author contributions

CZ and YL designed the research. CZ and JC performed the experiments. CZ and HL analyzed the data. CZ, JC, and YL wrote the manuscript. CZ, HL, XP, and YL revised the manuscript. All authors contributed to the article and approved the submitted version.

## Funding

This work was supported by Key Research and Development of Shandong Province, China (2019GNC106085) and A Project of Shandong Province Higher Educational Science and Technology Program, China (J18KA140).

## Conflict of interest

The authors declare that the research was conducted in the absence of any commercial or financial relationships that could be construed as a potential conflict of interest.

## Publisher’s note

All claims expressed in this article are solely those of the authors and do not necessarily represent those of their affiliated organizations, or those of the publisher, the editors and the reviewers. Any product that may be evaluated in this article, or claim that may be made by its manufacturer, is not guaranteed or endorsed by the publisher.
