# Supplementary material for: Sigma factor RpoS positively affects the spoilage activity of Shewanella baltica and negatively regulates its adhesion effect
Source: Front Microbiol. 2022 Sep 2;13:993237. doi: 10.3389/fmicb.2022.993237 (PMC9478337; doi:10.3389/fmicb.2022.993237)
Supplement: Supplementary file 1 [file Table_1.DOCX]

**Table S1**. Quality descriptions and scores for sensory evaluation of yellow croaker fillets.

| Quality attributes | Description | Score |
| --- | --- | --- |
| Colour | Fresh, creme white | 3 |
|  | Slightly yellowish | 2 |
|  | Yellow, brown, discolored | 1 |
| Texture | The muscle tissue is compact and complete with clear texture | 3 |
|  | The muscle tissue is not tight, but it's not loose. | 2 |
|  | Muscle tissue is not tight, loose. | 1 |
| Odour | Inherent fragrance, fresh. | 4 |
|  | Inherent fragrance, better fresh. | 3 |
|  | The inherent fragrance disappears. | 2 |
|  | There is a fishy smell or ammonia smell | 1 |
