# Supplementary material for: Sigma factor RpoS positively affects the spoilage activity of Shewanella baltica and negatively regulates its adhesion effect
Source: Front Microbiol. 2022 Sep 2;13:993237. doi: 10.3389/fmicb.2022.993237 (PMC9478337; doi:10.3389/fmicb.2022.993237)
Supplement: Supplementary file 2 [file Table_2.DOCX]

**Table S2**. The primers used in this study.

| primers | Relevant characteristics |
| --- | --- |
| 16S rRNA-F | 5′- TCGAGCGCGTACAACAGAAC -3′ |
| 16S rRNA-F | 5′- CCACCTTGCCACCATGAGTC -3′ |
| RS37220-F | 5′- CGCTAACTGGTTCCTCG -3′ |
| RS37220-R | 5′- TTAGTGACCGCTCGCTTC -3′ |
| RS42925-F | 5′- CGAAACCTGCGTAGGAGAA -3′ |
| RS42925-R | 5′- GATGTAGAACACCCGTGGC -3′ |
| RS34240-F | 5′- TTTTGTCTTCGCTGGTCG -3′ |
| RS34240-R | 5′- AATGCCCTTGGCTTTCG -3′ |
| RS32515-F | 5′- ATTGGGTAGAAGACCGAGAT -3′ |
| RS32515-R | 5′- ATACGCCCTTGGGATTAC -3′ |
