# Supplementary material for: Sigma factor RpoS positively affects the spoilage activity of Shewanella baltica and negatively regulates its adhesion effect
Source: Front Microbiol. 2022 Sep 2;13:993237. doi: 10.3389/fmicb.2022.993237 (PMC9478337; doi:10.3389/fmicb.2022.993237)
Supplement: Supplementary file 4 [file Table_4.DOCX]

**Table S4.** Alignment statistics of *S.baltica* and *rpoS* mutant mapped to the reference genome.

| Strain | Biologicalreplicates | Total  reads | Total mapped  reads | Percentage of Total mapped reads | Unique match | Muti-position matches |
| --- | --- | --- | --- | --- | --- | --- |
| 1. *baltica* wild type | 1 | 7,147,400 | 6,023,736 | 84.28% | 4,911,115 | 1112621 |
|  | 2 | 7,907,626 | 6,838,400 | 86.48% | 5,603,960 | 1234440 |
|  | 3 | 7,861,596 | 6,788,707 | 86.35% | 5,476,010 | 1312697 |
| *rpoS* mutant | 1 | 7,504,454 | 6,444,761 | 85.88% | 5,075,626 | 1369135 |
|  | 2 | 7,071,144 | 6,192,154 | 87.57% | 4,974,929 | 1217225 |
|  | 3 | 7,532,148 | 6,371,866 | 84.6% | 5,069,542 | 1302324 |
