# Supplementary material for: Sigma factor RpoS positively affects the spoilage activity of Shewanella baltica and negatively regulates its adhesion effect
Source: Front Microbiol. 2022 Sep 2;13:993237. doi: 10.3389/fmicb.2022.993237 (PMC9478337; doi:10.3389/fmicb.2022.993237)
Supplement: Supplementary file 8 [file Table_8.DOCX]

**Supplementary Table 1.** Quality descriptions and scores for sensory evaluation of yellow croaker fillets.

**Supplementary Table 2.** The primers used in this study.

**Supplementary Table 3.** Summary of reads in *S.baltica* and *rpoS* mutant transcriptome sequencing.

**Supplementary Table 4.** Alignment statistics of *S.baltica* and *rpoS* mutant mapped to the reference genome.

**Supplementary Table 5.** Detailed data related to the 397 DEGs between the *rpoS* mutant and the *S.baltica* wild-type strain.

**Supplementary Table 6.** Detailed data related to Go annotations.

**Supplementary Table 7.** Detailed data related to KEGG annotations.
